# Regulation of reactive oxygen species and phytohormones in osmotic stress tolerance during seed germination in *indica* rice

**DOI:** 10.3389/fpls.2023.1186960

**Published:** 2023-06-13

**Authors:** Ryusuke Kawaguchi, Chetphilin Suriyasak, Ryo Matsumoto, Yuta Sawada, Yuki Sakai, Norimitsu Hamaoka, Kazuhiro Sasaki, Koji Yamane, Yoichiro Kato, Christophe Bailly, Yushi Ishibashi

**Affiliations:** ^1^ Graduate School of Bioresource and Bioenvironmental Sciences, Kyushu University, Fukuoka, Japan; ^2^ Faculty of Agriculture, Kyushu University, Fukuoka, Japan; ^3^ Graduate School of Agricultural and Life Science, University of Tokyo, Tokyo, Japan; ^4^ Biological Resources and Post-harvest Division, Japan International Research Center for Agricultural Sciences, Tsukuba, Japan; ^5^ Graduate School of Agriculture, Kindai University, Nara, Japan; ^6^ Biologie des Semences, Unité Mixte de Recherche (UMR) 7622, The Institut de Biologie Paris-Seine (IBPS), Sorbonne Université, Paris, France

**Keywords:** germination, reactive oxygen species, gibberellic acid, abscisic acid, osmotic stress, rice

## Abstract

Climate change due to global warming is now affecting agricultural production worldwide. In rice, one of the most important crops, water limitation due to irregular rainfall in rainfed lowlands during crop growth limits yield. Dry direct-sowing has been proposed as a water-efficient approach to cope with water stress during rice growth, but poor seedling establishment due to drought during germination and emergence is a problem. Here, we germinated *indica* rice cultivars Rc348 (drought tolerant) and Rc10 (drought sensitive) under osmotic stress induced by PEG to elucidate mechanisms of germination under drought. Rc348 had higher germination rate and germination index under severe osmotic stress of −1.5 MPa, above those of Rc10. Rc348 showed up-regulated GA biosynthesis, down-regulated ABA catabolism, and up-regulated α-amylase gene expression in imbibed seeds under PEG treatment compared to that of Rc10. During germination, reactive oxygen species (ROS) play important roles in antagonism between gibberellic acid (GA) and abscisic acid (ABA). Embryo of Rc348 treated with PEG had significantly greater expression of NADPH oxidase genes and higher endogenous ROS levels, together with significantly increased endogenous GA_1_, GA_4_ and ABA contents compared to that of Rc10. In aleurone layers treated with exogenous GA, expression of α-amylase genes was higher in Rc348 than in Rc10, and expression of NADPH oxidase genes was enhanced with significantly higher ROS content in Rc348, suggesting higher sensitivity of GA to ROS production and starch degradation in aleurone cells of Rc348. These results suggest that the osmotic stress tolerance of Rc348 is due to enhancement of ROS production, GA biosynthesis, and GA sensitivity, resulting in a higher germination rate under osmotic stress.

## 1 Introduction

Rice (*Oryza sativa* L.) is one of the most important staple crops, feeding a third of the world’s population. It is produced mainly in Asia, largely in flooded conditions. Increasing demand for rice production and diminishing rainfall due to climate change profoundly affect rice production, for which reliable irrigation is crucial (Vries et al., 2010). Rainfed lowland covers more than 30% of rice cultivation areas globally and in major rice producing countries (Matloob et al., 2015; Gadal et al., 2019). However, weather fluctuations due to climate change in recent years and the irregular rainfall in rainfed lowlands, often delay transplantation (Ohno et al., 2018). Prolonged water stress during the transition from vegetative stage to reproductive stage delays heading and significantly reduces yield (Pantuwan et al., 2002). To cope with water stress in rice, dry direct-sowing (DDS) has been proposed as a water-efficient approach, since it uses much less water than transplantation into puddled fields (Haefele et al., 2016). DSS method has been adopted in many countries (Shekhawat et al., 2020), which more than 25% of total rice production in tropical regions in Asia, and more than 90% of rice cultivated areas in the United States and Sri Lanka depend on DDS (Kumar and Ladha, 2011; Subedi et al., 2019). DDS is a promising approach for rainfed rice cropping, using less labor and having no need for irrigation or seedling preparation (Hayashi et al., 2007; Kato and Katsura, 2014). However, it faces problems of weed infestation and poor seedling establishment if drought occurs during germination and emergence (Yamane et al., 2017; Ohno et al., 2018).

Germination is a crucial developmental stage and is regulated by many factors, including the phytohormones gibberellic acid (GA), which induces germination, and abscisic acid (ABA), which suppresses germination (Liu et al., 2010; Ishibashi et al., 2012; Jacobsen et al., 2020). Biosynthesis of GA involves many catalytic enzymes, including ent-kaurene acid oxidase (KAO), GA 20-oxidase (GA20ox), and GA 3-oxidase (GA3ox) (Hedden and Phillips, 2000). ABA is synthesized by the enzyme 9-cis epoxycarotenoid dioxygenase (NCED) and is biodegraded by a cytochrome P450 monooxygenase or ABA 8’-hydroxylase *(ABA8′OH)* (Millar et al., 2006). Reactive oxygen species (ROS) as developmental and stress-signaling molecules are also involved *via* an ‘oxidative window’, wherein ROS homeostasis regulates germination (Bailly et al., 2008). ROS produced by NADPH oxidases during seed imbibition induce the production of GA and inhibit ABA to promote germination, which ROS homeostasis is important for abiotic stress responses *via* phytohormone signaling in many species (Oracz et al., 2007; Bailly et al., 2008; Ishibashi et al., 2010; Ishibashi et al., 2012; Ye et al., 2012; El-Maarouf-Bouteau et al., 2015; Shi et al., 2020; Wu et al., 2020). To degrade stored starch, the production of α-amylases, starch-hydrolyzing enzymes, are regulated by GA and ABA signaling factors such as GAMYB (GA-induced MYB-like transcription factor) and PKABA (ABA-induced protein kinase ABA-responsive protein kinase) which induces and inhibits expression of α-amylases, respectively, in cereal aleurone layers (Gubler et al., 1999; Gomez-Cadenas et al., 2001; Kaneko et al., 2002; Woodger et al., 2003; Ishibashi et al., 2012). Under osmotic stress caused by polyethylene glycol (PEG), α-amylase activity is inhibited, and germination is impaired (Bialecka and Kepczynski, 2010; Muscolo et al., 2013).

*Indica* rice Rc348 is a newly released drought-tolerant DDS cultivar that has a higher seedling emergence rate than the common and widely grown drought-sensitive cultivar Rc10, resulting in higher yield under drought stress on farm experiments in the Philippines (Yamane et al., 2017; Ohno et al., 2018). Both germination ability and seedling establishment are crucial for later growth and development (Yamane et al., 2017; Ohno et al., 2018). Although many studies have suggested drought-tolerant traits and cultivars for DDS cropping, the molecular mechanisms underlying drought responses of tolerant cultivars, especially in germination, are not yet well studied.

Here, we focused on germination ability of Rc348 under osmotic stress. We aimed at elucidating how different rice cultivars respond to osmotic pressure, an important component of drought stress, at the transcriptional, hormonal, and ROS levels during seed imbibition.

## 2 Materials and methods

### 2.1 Plant materials and growth conditions

Three-week-old seedlings of *indica* rice (*Oryza sativa* L.) cvv. Rc348, Rc10, Rc420, Rc222, and Dular were transplanted into 1/2000-a Wagner pots (5 plants per pot) with 32.8 g of basal dressing compound fertilizer (N–P–K: 4%–4%–4%) and 3.2 g of sigmoid-type controlled-release coated urea. Topdressing of 1.88 g of ammonium sulfate (21% N) per pot was applied during the tiller development stage and the panicle booting stage. Plants were grown under natural conditions at Kyushu University, Fukuoka, Japan, from mid-May to late-October in 2019. Anthesis, the day when spikelets on the upper primary rachis branches flowered on >50% of the population, was set as the day of flowering (0 DAF; days after flowering). Plants were harvested at 49 DAF. Harvested seeds were dried at room temperature for 1 week and stored at −30°C to maintain dormancy. Seed morphology of all cultivars are shown in **Supplemental Figure 1**.

### 2.2 Seed germination test under osmotic stress and exogenous chemical treatments

Seeds of all cultivars underwent dormancy break treatment at 45°C in the dark for 2 weeks to ensure a uniform degree of seed dormancy. Seeds were rested at room temperature for 1 h, sterilized in 0.2% NaClO for 20 min and washed thoroughly in sterilized distilled water; 30 seeds were placed in 9-cm Petri dishes with 10 mL of sterilized distilled water (control) or −0.5, −1.0, or −1.5 MPa of PEG 4000 solution (Nacalai Tesque inc., Kyoto, Japan) to germinate at 28°C in the dark. Germination rates were recorded every 6 h until 144 h after imbibition (HAI). A seed was recorded as germinated when shoot length was ≥0.2 cm. The germination index of each sample was calculated as described by Coolbear and Grierson (1984).

In the experiment with exogenous GA and ABA, embryoless half-seeds were imbibed in 10 mL of 1 µM GA_3_ in −1.5-MPa PEG solution on a filter paper in a Petri dish at 28°C in the dark, with or without 5 µM ABA, and transcript levels of *GAMYB, SAPK*, α-amylase, and NADPH oxidase genes were analyzed at 24 HAI and endogenous ROS content was measured at 36 HAI.

In the GA sensitivity experiment, embryoless half-seeds were imbibed in 10 mL of 1 µM GA_3_ in −1.5-MPa PEG solution on a filter paper in a Petri dish at 28°C in the dark and sampled at 36 HAI for endogenous hydrogen peroxide content measurement.

In the experiment with exogenous sodium ascorbate, seeds were imbibed in 10 mL of -1.5 MPa PEG or 5, 15 and 25 mM of sodium ascorbate dissolved in -1.5MPa PEG solution. Germination percentage, gene expression and endogenous hormonal levels were analyzed at 84 HAI.

Seeds were imbibed with 6 mL of -1.5 MPa PEG supplied with exogenous 100 μM Diphenyleneiodonium chloride (DPI) or 10, 20, and 50 mM H_2_O_2_ with equal amount of DMSO to that of DPI solution. Germination rates were recorded with the same methods above.

### 2.3 RNA extraction and quantitative real-time PCR analysis

Total RNA from whole seeds, embryos, and embryoless half-seeds was extracted from frozen materials by the SDS/phenol/LiCl method (Chirgwin et al., 1979). cDNA was synthesized from extracted RNA with ReverTra Ace reverse transcriptase (Toyobo co., Ltd., Osaka, Japan) according to the manufacturer’s instructions. Quantitative real-time PCR was performed on a CFX Connect Optics Module Real-time PCR detector system (Bio-Rad) with SYBR Green dye (Toyobo) as described in the manufacturer’s instructions. PCR thermal cycling conditions were as follows: initial denaturation at 94°C for 2 min; 40 cycles of denaturation at 94°C for 20 s, annealing at a primer-specific temperature for 20 s (**Table S1**), and extension at 72°C for 20 s; followed by melting and plate reading. The data were normalized to the expression of *OsActin*.

### 2.4 Endogenous GA and ABA contents

Endogenous GA_1_, GA_4_, and ABA contents in embryos imbibed in −1.5-MPa PEG at 72 HAI were analyzed by LC-MS/MS (Exion LC and X500B, AB Sciex) as described by Xin et al. (2020). Three biological replicates were measured, each comprising embryos from 300 seeds. Isotope internal standards of GA_1_, GA_4_, and ABA were purchased from OlChemIm (Olomouc, Czech Republic).

### 2.5 NADPH oxidase enzyme activity

Embryos of 30 seeds from Rc348 and Rc10 (−1.5 MPa PEG at 48 HAI) were ground into fine power with liquid nitrogen. Ice cold 2 mL of Na-phosphate buffer (pH 8.0) was added to the sample and the contents were mixed and sonicate for 15 s prior to centrifugation at 16,000 *g* for 15 min at 4°C. Crude embryo homogenates were precipitated with acetone (9:1, acetone:homogenate) at −30°C for 15 min. Precipitated proteins were collected from centrifugation at 12,500 rpm for 10 min at 4°C. Protein pallets were resuspended in reaction buffer (50 mM Tris-HCl pH 8.0, 0.1 mM MgCl_2_, 0.25 M sucrose and 0.1% Triton-X100) and used for enzyme activity assay. The reaction of NADPH-dependent superoxide generation was measured using NBT (nitro blue tetrazolium chloride) at 530 nm in a spectrophotometer (Gynesys 40, Thermofisher Scientific) as previously described (Van Gestelen et al., 1997; Sarath et al., 2007; Ishibashi et al., 2010). Monoformazan concentrations were calculated using an extinction coefficient of 12.8 mM^-1^ cm^-1^.

### 2.6 Endogenous hydrogen peroxide content

Embryos of 20 seeds imbibed in −1.5-MPa PEG and embryoless half-seeds imbibed in 1 µM GA_1_ in −1.5-MPa PEG were sampled at 24 and 36 HAI, respectively. Samples were snap-frozen in liquid nitrogen and stored at −80°C before analysis. Samples were homogenized in 2 mL of 0.2 M perchloric acid on ice and centrifuged at 13,000 rpm at 4°C for 15 min. Supernatant (0.5 mL) was mixed with 0.5 mL of 4 M KOH, and samples were centrifuged at 1000× *g* at 4°C for 5 min. The H_2_O_2_ content was measured by peroxidase-based assay as described by Ishibashi et al. (2015) and O’Kane et al. (1996).

### 2.7 Statistical analysis

Statistical analyses in this study were performed in SPSS statistical software version 28.0.0.0 (IBM). Differences among treatments were analyzed by one-tailed Student’s *t*-test and Tukey’s test with biological replications described in figure legends.

## 3 Results

### 3.1 Delayed germination under osmotic stress

**Figure 1:**
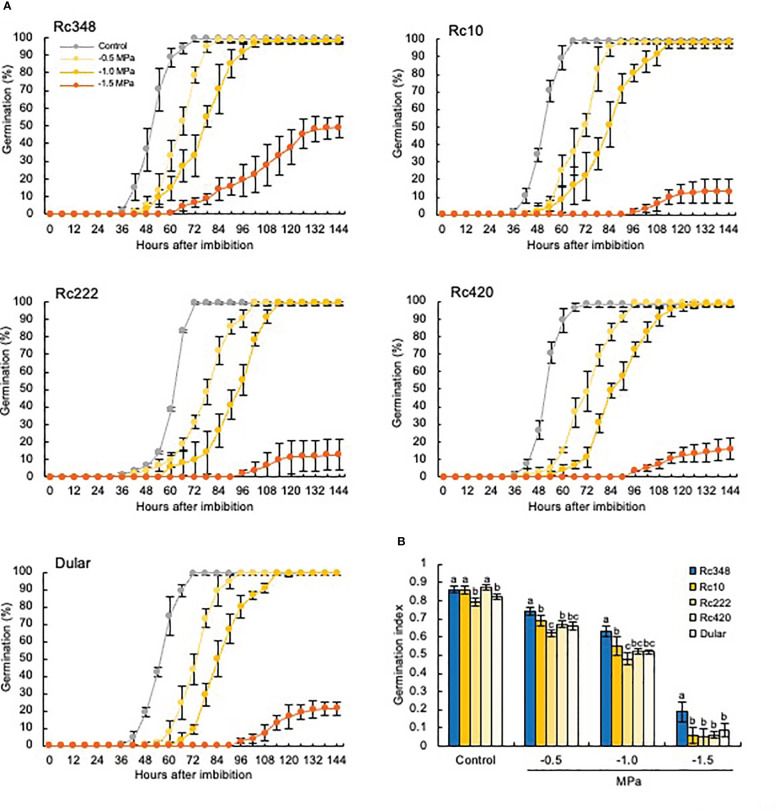
**(A)** Germination rates and **(B)** germination index of five *indica* rice cultivars under osmotic stress imposed by PEG. Control, germination in distilled water. Values with the same letter within a treatment are not significantly different at *P* < 0.05 by Tukey’s test (*n =* 5).

Imbibition of seeds of all five cultivars in PEG suppressed germination in a concentration-dependent manner (**Figure 1A**). Under severe osmotic pressure of −1.5 MPa, Rc348 had the fastest germination and the highest final germination rate of about 50%, whereas those of the other cultivars were ≤20%. Rc348 had a significantly higher GI than the other cultivars at all PEG concentrations, and about double that of the other cultivars at −1.5 MPa (**Figure 1B**). Therefore, we used this PEG concentration in all other experiments.

### 3.2 Expression of GA and ABA related genes in imbibed seeds under osmotic stress

**Figure 2:**
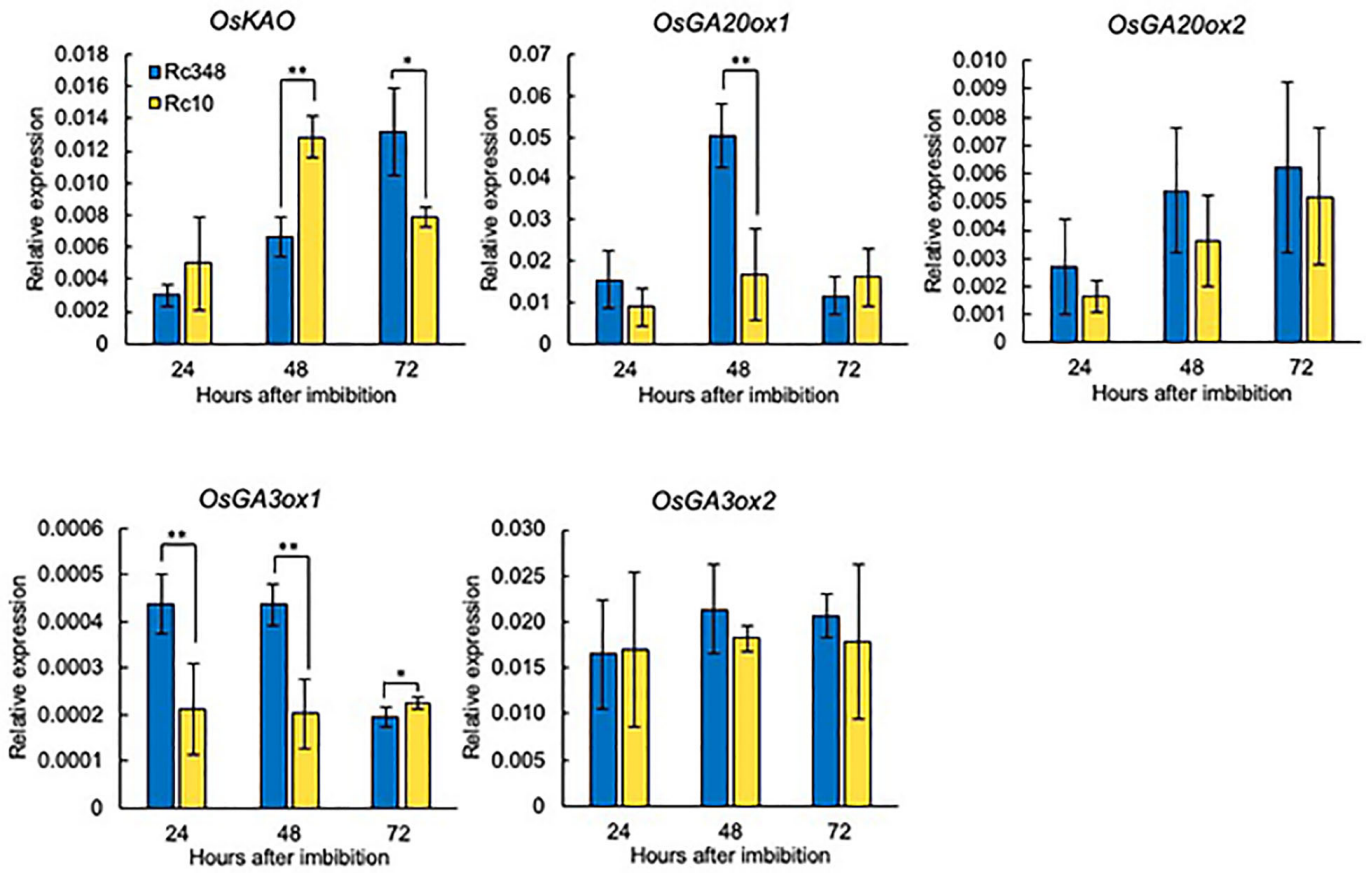
Relative expression of GA biosynthesis genes at 24, 48, and 72 HAI during seed imbibition under osmotic stress imposed by −1.5 MPa PEG. Significant differences: *P < 0.05, **P < 0.01 by Student’s *t-*test (*n =* 3).

As a reference, we chose the widely grown drought-tolerant cultivar Rc348 and drought-sensitive cultivar Rc10 within all examined cultivars (**Supplemental Figure 1**). We analyzed transcript levels of GA- and ABA-metabolism-related genes and contents of GA and ABA in seeds during imbibition at 24, 48 and 72 HAI. Among genes for GA biosynthesis, despite the significantly lower expression of *OsKAO* in Rc348 at 48 HAI (1/1.9×), and no significant difference in *OsGA3ox2* expression, Rc348 had significantly higher *OsGA20ox1* expression at 48 HAI (3.0× that of Rc10), and marginally higher at 24 HAI. Significantly higher *OsGA3ox1* expression of Rc348 compared to that of Rc10 at 24 HAI (2.1×) and 48 HAI (2.1×) was also observed (**Figure 2**). Transcript levels of *OsGA3ox2* remained stable and showed no significant difference over time in both cultivars.

**Figure 3:**
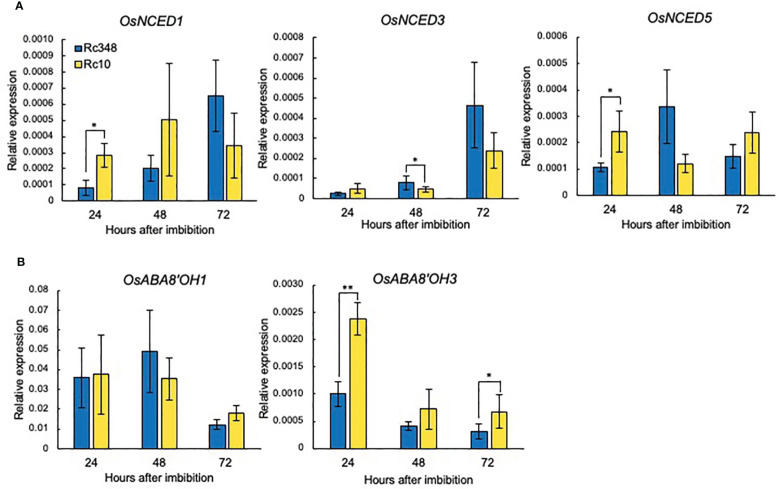
Expression of **(A)** ABA biosynthesis and **(B)** ABA catabolism genes at 24, 48, and 72 HAI during imbibition under osmotic stress imposed by −1.5 MPa PEG. Significant differences: *P < 0.05, **P < 0.01 by Student’s *t-*test (*n =* 3).

Among genes for ABA biosynthesis (*OsNCED*s), *OsNCED1* and *OsNCED3* expression gradually increased from 24 to 72 HAI in both Rc10 and Rc348 under osmotic stress. On the other hands, changes of *OsNCED5* expression overtime from 24 to 72 HAI were barely observed. Rc10 had significantly higher *OsNCED1* and *OsNCED5* expression at 24 HAI. Rc348 showed higher expression of *OsNCED3* and *OsNCED5* at 48 HAI, together with marginally higher expression of *OsNCED1* and *OsNCED3* at 72 HAI (**Figure 3A**). Despite no change in *OsABA8’OH1* expression, Rc348 had significantly lower *OsABA8’OH3* expression at 24 HAI (1/2.4×) and 72 HAI (1/2.2×). Overall, with fluctuations during germination time course of ABA biosynthesis genes, significant downregulation of *OsABA8’OH3* for ABA catabolism in Rc348 was observed (**Figure 3B**).

### 3.3 NADPH oxidase gene expression, ROS and hormone contents in embryos under osmotic stress

**Figure 4:**
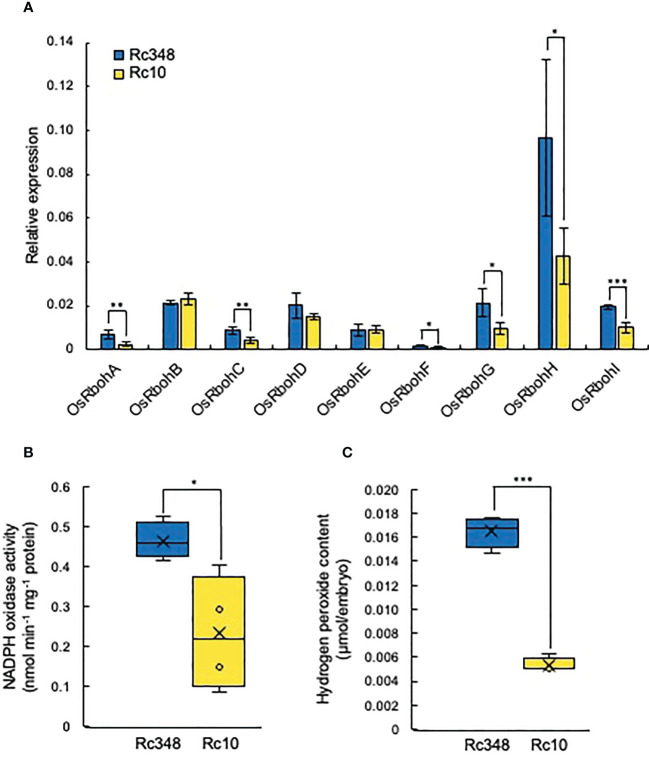
Expression of rice NADPH oxidases, NADPH oxidase enzyme activity, and endogenous hydrogen peroxide content in embryos in −1.5 MPa PEG. **(A)** Relative expression of *OsRbohA* to *OsRbohI* at 48 HAI. **(B)** Endogenous NADPH oxidase activity and **(C)** hydrogen peroxide content in embryo at 48 HAI. Significant differences: *P < 0.05, **P < 0.01, ***P < 0.001 by Student’s *t-*test [*n =* 3 for A, *n =* 4 for (**B**, **C**)].

Since GA and ABA are known to be regulated by ROS in embryos, we then analyzed the transcript levels of nine *NADPH oxidase* genes (*Respiratory burst oxidase homologs*, *OsRboh*s) in embryos during imbibition in −1.5-MPa PEG at 48 HAI (**Figure 4A**). Expression of *OsRbohA*, *OsRbohC*, *OsRbohF*, *OsRbohG*, *OsRbohH*, and *OsRbohI* was significantly higher in Rc348 than in Rc10 (**Figure 2A**); *OsRbohH* had the highest transcript level in *OsRboh*s (2.8× that in Rc10). NADPH oxidase activity in embryos of Rc348 was also 2.0× significantly higher than that in Rc10 (**Figure 4B**), resulting in significantly enhanced endogenous ROS content in Rc348 embryos for 3.1× that in Rc10 (**Figure 4C**). These results show that osmotic stress enhanced NADPH oxidase gene expression and increased ROS content in Rc348 embryos. We also showed that inhibition of NADPH oxidase by DPI significantly reduced germination rate of Rc348, where exogenous H_2_O_2_ significantly improved Rc10 seed germination under −1.5-MPa PEG (**Supplemental Figure 2**), suggesting the role of ROS on seed germination under osmotic stress. We also analyzed endogenous GA_1_, GA_4_ and ABA in imbibed embryos at 72 HAI. Rc348 had significantly higher content of endogenous GA_1_ (1.9×), GA_4_ (1.9×), and ABA (2.0×) than Rc10, which is explained by upregulated GA biosynthesis and downregulated ABA catabolism transcript levels during imbibition in Rc348 seeds under osmotic stress (**Table 1**). Since enhanced endogenous ROS stimulated GA production without decreasing ABA content to promote germination in Rc348, exogenous sodium ascorbate (AsA), an antioxidant to decrease endogenous ROS, was applied to elucidate the role of ROS on GA and ABA production under osmotic stress in Rc348 (**Supplemental Figure 3**). As a result, exogenous AsA significantly reduced germination rate in dose dependent manner under −1.5 MPa PEG (**Supplemental Figure 1A**). Despite no obvious change in ABA metabolism gene expression, Rc348 seeds imbibed with 25 mM AsA showed significantly reduced expression of GA biosynthesis, *OsGA20ox1* (1/4.3×) and *OsGA3ox2* (1/33.8×) compared to that of −1.5 MPa PEG only during imbibition (**Supplemental Figure 3B−C**). Consequently, endogenous GA_1_ content was significantly reduced by exogenous AsA, while ABA and GA_4_ content remained unchanged (**Supplemental Figure 3D−F**). Thus, these results suggest that enhancement of ROS rather induce GA production than inhibiting ABA to promote seed germination in Rc348 under osmotic stress.

**Table 1:** Endogenous GA_1_, GA_4_, and ABA contents in imbibed seeds under osmotic stress imposed by −1.5 MPa PEG.

Cultivar	GA_1_ content(pg/seed)	GA_4_ content (pg/seed)	ABA content (pg/seed)
Rc348	0.185 ± 0.053	0.550 ± 0.129	8.840 ± 1.824
Rc10	0.099 ± 0.037	0.293 ± 0.114	4.391 ± 2.134
Student’s *t*-test (n=3)	*P=*0.046*	*P=*0.031*	*P=*0.026*

Values are means ± SD of 3 biological replicates. Significant differences by Student’s t-test.

### 3.4 α-Amylase gene expression in imbibed seeds under osmotic stress

**Figure 5:**
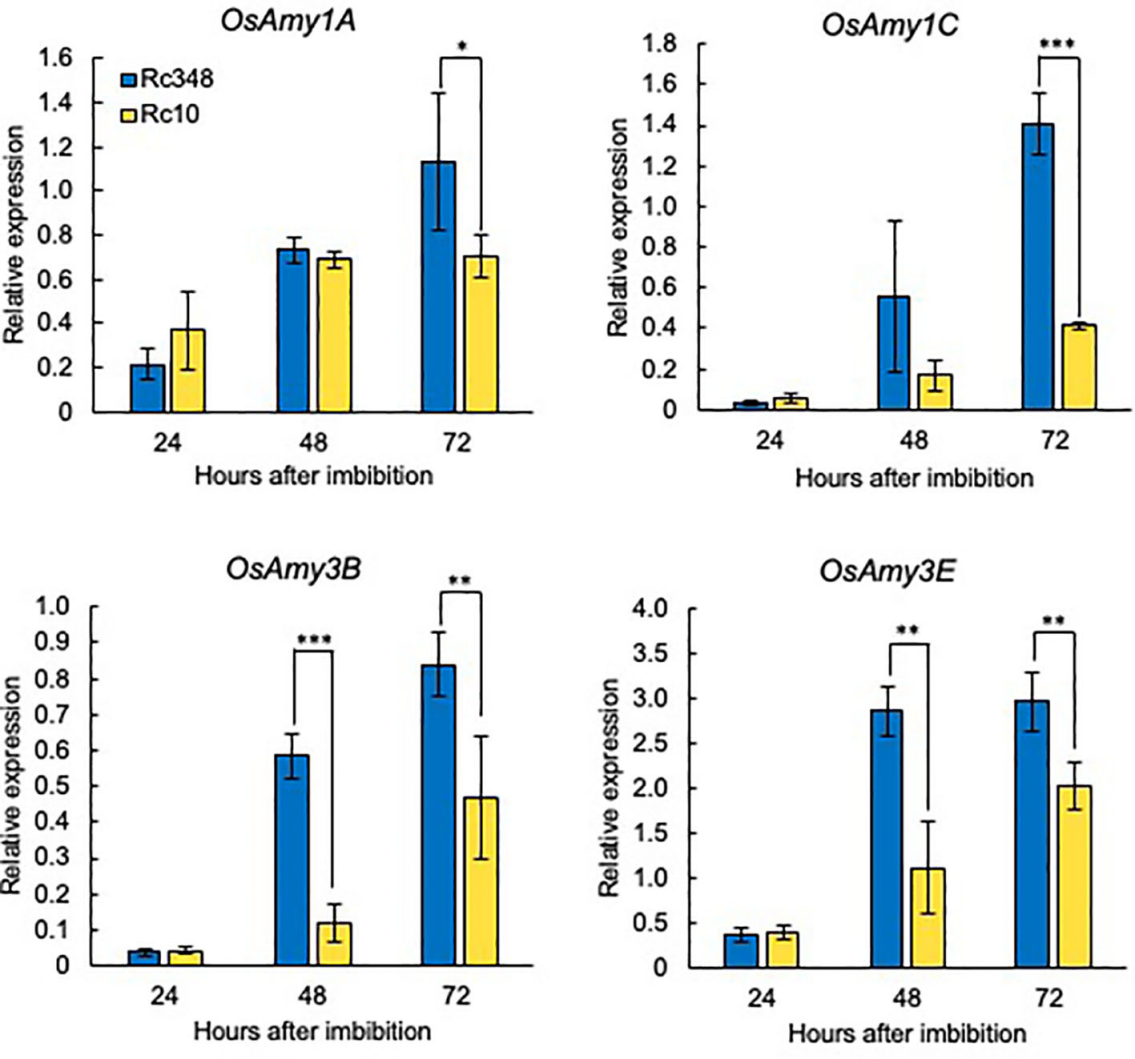
Relative expression of α-amylase genes at 24, 48, and 72 HAI during imbibition under osmotic stress imposed by −1.5 MPa PEG. Significant differences: *P < 0.05, **P < 0.01, ***P < 0.001 by Student’s *t-*test (*n =* 3).

α-Amylase is induced by GA and suppressed by ABA in cereal aleurone cells (Woodger et al., 2003). During seed imbibition, the expression of α-amylase genes (*OsAmy1A*, *OsAmy1C*, *OsAmy3B*, and *OsAmy3E*) is induced by GA, and they are highly expressed in rice endosperm after imbibition (Chen, 2006). We analyzed the expression of these genes in imbibed seeds during germination under osmotic stress (**Figure 5**). Rc348 had significantly higher α-amylase gene expression than Rc10 at 48 HAI (*OsAmy3B*, 4.8×; *OsAmy3E*, 2.6×) and 72 HAI (*OsAmy1A*, 1.6×; *OsAmy1C*, 3.4×; *OsAmy3B*, 1.8×; *OsAmy3E*, 1.5×). These results suggest that α-amylase upregulation during imbibition of Rc348 seeds facilitates germination under osmotic stress.

### 3.5 Responses of starch degradation and ROS accumulation in aleurone cells to exogenous GA and ABA

**Figure 6:**
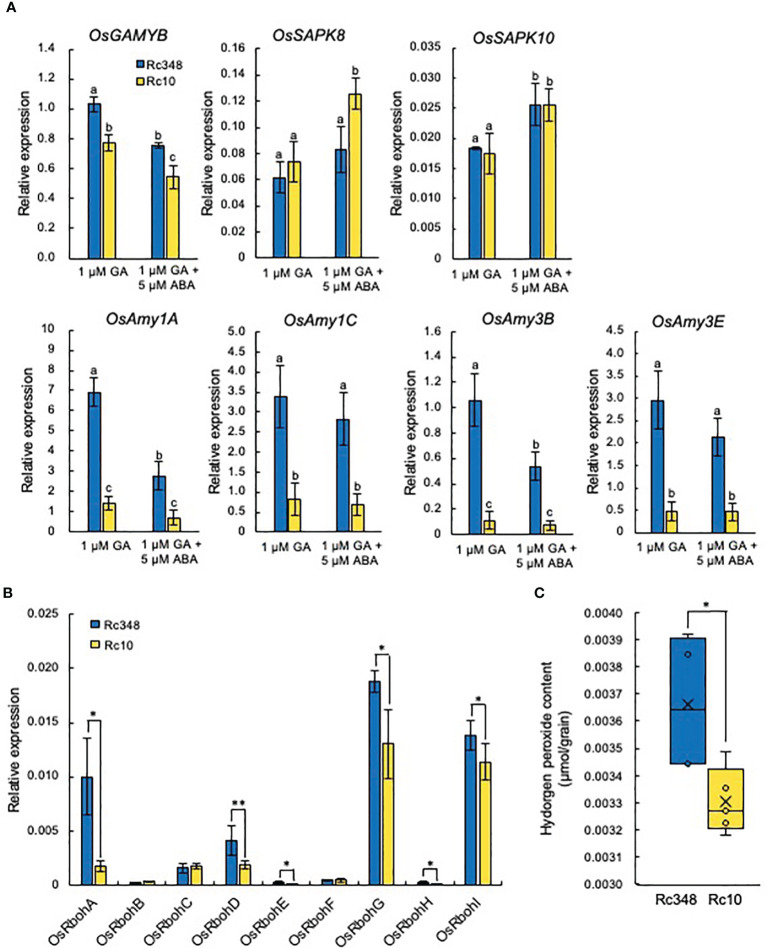
Induction of GA and ABA signaling and NADPH oxidase genes in aleurone layers under osmotic stress. **(A)** Relative expression of *OsGAMYB*, *OsSAPK8,10*, and α-amylase genes at 24 HAI in aleurone layers of embryoless seeds in −1.5 MPa PEG + 1 µM GA or −1.5 MPa PEG + 1 µM GA + 5 µM ABA. **(B)** Relative expression of NADPH oxidase genes at 24 HAI in −1.5 MPa PEG + 1 µM GA. **(C)** Endogenous hydrogen peroxide content in aleurone layers of embryoless seeds at 36 HAI in −1.5 MPa PEG + 1 µM GA. In A, values with the same letter are not significantly different at *P* < 0.05 by Tukey’s test (*n =* 3). **(B, C)** Significant differences: *P < 0.05, **P < 0.01 by Student’s *t*-test (*n =* 3 for **A**, **B**, *n* = 4 *for* **C**).

Rc348 imbibed seeds had significantly higher expression of α-amylase genes (**Figure 5**). In aleurone cells, the expression of GAMYB and its downstream target α-amylase is induced by GA and suppressed by ABA through PKABA induction (Gomez-Cadenas et al., 2001; Woodger et al., 2003; Ishibashi et al., 2012). It has been shown that rice SAPK8 and SAPK10 of SAPK family genes in rice are orthologous to PKABA1 in barley, which expression of both is induced by ABA (Li et al., 2007). We investigated the effects of exogenous GA with/without of ABA on GAMYB, PKABA and α-amylase gene expression in aleurone cells at 24 HAI (**Figure 6**). Exogenous GA alone significantly increased expression of Os*GAMYB* (1.3×) relative to level in Rc10 (**Figure 6A**). Presence of exogenous ABA inhibited the expression of *OsGAMYB* in both Rc348 and Rc10, however, the expression was significantly increased in Rc348 (1.4×) relative to levels in Rc10. Exogenous ABA induced the expression of *OsSAPK8* and *OsSAPK10* in aleurone cells. Despite no change between Rc348 and Rc10 in OsSAPK10 expression, Rc348 showed significantly reduced expression of *OsSAPK8* (1/1.5×) compared to level in RC10. Exogenous GA alone significantly increased expression of α-amylase genes (*OsAmy1A*, 4.9×; *OsAmy1C*, 4.2×; *OsAmy3B*, 9.5×; *OsAmy3E*, 6.1×) relative to levels in Rc10 (**Figure 6A**). α-amylase gene expression was also increased by GA even in the presence of ABA in Rc348 (3.9×, 4.1×, 7.3×, and 4.6×, respectively). Therefore, the suppressive effect of ABA was countered by the inductive effect of GA on signaling and starch degradation in aleurone cells of Rc348. GA induces *RboH* gene expression to regulate α-amylase activity in barley aleurone cells (Ishibashi et al., 2015). We analyzed the transcript levels of NADPH oxidase genes in aleurone cells treated with exogenous GA and found that *OsRbohA*, *OsRbohD*, *OsRbohE*, *OsRbohG*, and *OsRbohI* expression (**Figure 6B**) and endogenous ROS levels in aleurone cells (**Figure 6C**) were significantly higher in Rc348 than in Rc10. These results suggest that Rc348 also had higher sensitivity to exogenous GA in terms of ROS induction in aleurone cells.

## 4 Discussion

We propose that Rc348, a newly developed drought-stress-tolerant cultivar bred for DDS (Yamane et al., 2017; Ohno et al., 2018), gains its capacity for a high germination rate under osmotic stress *via* the regulation of ROS and phytohormones. Loss of function of NADPH oxidase of *osrbohb* mutant results in reduced osmotic stress tolerance due to lower levels of ROS and ABA contents in rice seedlings and resulted in impaired seed germination (Shi et al., 2020). In plants, *Rboh* genes not only function in responses to stress signaling and development (Kaur and Pati, 2016; Suriyasak et al., 2017; Chapman et al., 2019), but also promote germination (Muller et al., 2009; Ishibashi et al., 2010; Ishibashi et al., 2015; Kai et al., 2016; Ishibashi et al., 2017). After imbibition, ROS produced in seeds induce GA and inhibit ABA production to initiate germination (Liu et al., 2010; Ishibashi et al., 2015). We showed that Rc348 had the highest ability to germinate under a severe osmotic stress of -1.5 MPa, when compared to other cultivars tested. ROS induce production of GA (which promotes germination) and inhibit production of ABA (which suppresses germination) (Oracz et al., 2007; Ishibashi et al., 2012; El-Maarouf-Bouteau et al., 2015; Ishibashi et al., 2015). In barley embryos treated with diphenylene iodonium chloride (DPI), an NADPH oxidase inhibitor, endogenous GA was significantly reduced while ABA was enhanced resulting in inhibited germination (Ishibashi et al., 2015). In our results, we observed increased ROS content together with higher endogenous GA_1_, GA_4_ and ABA contents in Rc348, which were due to up-regulated *OsGA20ox1*, *OsGA3ox1* and *OsNCED3*, and down-regulated *OsABA8’OH3*. For ABA biosynthesis, previous studies have reported that *OsNCED1* plays a role in salinity stress response (Zhang et al., 2022) and heat stress tolerance (Zhou et al., 2022), where *OsNCED3* expression is highly induced by PEG and other osmotic stresses, contributing to ABA accumulation for stress responses (Huang et al., 2018). Here, we observed overall increase in *OsNCED1* and *OsNCED3* expression overtime upon germination under osmotic stress in both cultivars. This suggests the possibility of ABA accumulation due to osmotic stress response in both cultivars. For *OsNCED5*, we could not observe obvious difference between cultivars from 24 to 72 HAI, which might be due to that its expression drops rapidly after imbibition and stays at the same basal level from 18 HAI onward (Suriyasak et al., 2020). Additionally, *OsABA8’OH3* expression in Rc348 was lower than that in Rc10 under osmotic stress. High endogenous ABA level in RC348 under osmotic stress might be attributed to the *OsABA8’OH3* expression. For GA biosynthesis, we did not observe change in *OsGA3ox2* expression over time, which might be due to its rapid peak at the very early stage of imbibition as reported in previous study (Kaneko et al., 2002), whereas *OsGA20ox1* expression peaks at the later phase of germination (Liu et al., 2014). A previous study has shown that expression of GA biosynthetic genes, including *OsGA20ox1* and *OsGA3ox1*, was suppressed by ABA in rice (Ye et al., 2012). In our study, enhancement of endogenous ABA did not inhibit expression of *OsGA20ox1* and *OsGA3ox1* in Rc348 under osmotic stress. Our results showed that enhanced ROS rather promote GA than act to suppress ABA in Rc348. To explain this phenomenon, we observed that exogenous sodium ascorbate (AsA), an antioxidant, significantly inhibited seed germination of Rc348 *via* suppressing GA biosynthesis genes, *OsGA20ox1* and *OsGA3ox2*, rather than affecting ABA metabolism genes (**Supplemental Figure 3A−C**). Consequently, endogenous GA_1_ level in seeds was not detected by AsA treatment, without affecting endogenous ABA and GA_4_ contents (**Supplemental Figure 3D−F**), which confirmed our results that enhancement of ROS mainly induces GA production to promote germination under osmotic stress of Rc348. Additionally, drought-tolerant maize and *Medicago sativa* L. seedlings accumulated more endogenous ABA in leaves under osmotic stress induced by PEG than drought-intolerant seedlings (Yao et al., 2019; Liu et al., 2022). Since ABA is known to accumulate under osmotic stress and enhance stress responses (Kuromori et al., 2018), in this study also, enhancement of ABA under osmotic stress in Rc348 might be involved in osmotic stress tolerance, with better seedling establishment under drought, as described in our previous study (Yamane et al., 2017).

**Figure 7:**
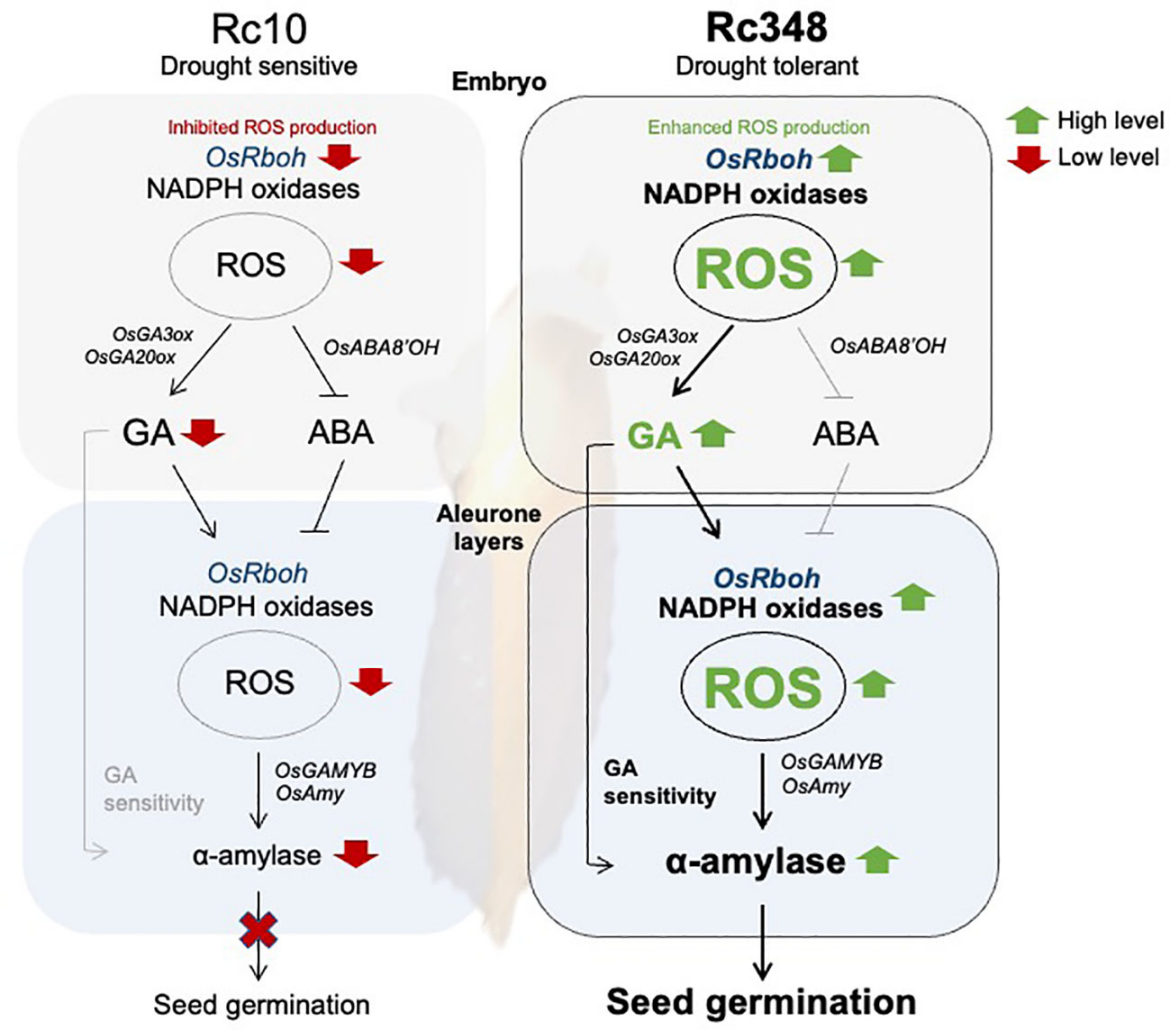
Scheme of regulations of reactive oxygen species in osmotic stress tolerance during seed germination suggested in this study. Pathways in black lines are shown to be more effective than that in grey lines in this study.

In cereal aleurone cells, GAMYB is a transcription factor that is upregulated by GA and downregulated by ABA (Gomez-Cadenas et al., 2001; Washio, 2003; Woodger et al., 2003; Ishibashi et al., 2012), and binds to GARE boxes in α-amylase promoters to induce starch degradation (Kaneko et al., 2002). Osmotic stress reduces α-amylase activity and thus impairs germination (Muscolo et al., 2013). Here, we showed that Rc348 gains its osmotic tolerance *via* upregulation of α-amylase gene expression in aleurone cells due to higher endogenous bioactive GA levels in embryos under osmotic stress. In aleurone cells of Rc348, expression of both *GAMYB* and α-amylase genes was highly induced by exogenous GA, suggesting its higher responses to GA than Rc10’s. In barley aleurone cells, PKABA induced by ABA inhibits *GAMYB* and α-amylase expression (Ishibashi et al., 2012). Here we showed that ABA induction of SAPK8 was significantly lower in Rc348 aleurone cells. Consequently, GAMYB and α-amylase induction in aleurone cells was still significantly higher in Rc348 than in Rc10 even in the presence of exogenous ABA, suggesting the importance of enhanced GA signaling in Rc348 for starch degradation to fuel germination. We previously showed that GA stimulates NADPH oxidase gene expression for ROS production in aleurone layer of barley seed, which inhibits PKABA to promote α-amylase expression (Ishibashi et al., 2012; Ishibashi et al., 2015). Rc348 also had higher sensitivity than Rc10 to exogenous GA in terms of higher *Rboh* expression and endogenous ROS content in aleurone cells, which consequently led to α-amylase induction in aleurone cells *via* up-regulation of GAMYB and down-regulation of PKABA. Despite an increase in endogenous ROS contents in both embryos and aleurone cells, expression patterns of *OsRboh* genes differed between the two: *OsRbohH* was expressed mainly in embryos, while *OsRbohA*, *OsRbohG*, and *OsRbohI* were highly expressed in GA-treated aleurone. In summary, the osmotic stress tolerance in seed germination of Rc348 is caused by enhancement of ROS production, GA biosynthesis, and GA sensitivity (**Figure 7**).

## Data availability statement

The original contributions presented in the study are included in the article/**Supplementary Material**. Further inquiries can be directed to the corresponding author.

## Author contributions

YK, KY, KS, CS, and YI designed the experiments; RK, CS, RM, YSaw, YSak, NH, and YI performed the experiments; RK, CS, and YI performed data analysis; CS, CB, and YI wrote the manuscript. All authors contributed to the article and approved the submitted version.
