# Supplementary material for: Regulation of reactive oxygen species and phytohormones in osmotic stress tolerance during seed germination in indica rice
Source: Front Plant Sci. 2023 Jun 13;14:1186960. doi: 10.3389/fpls.2023.1186960 (PMC10295146; doi:10.3389/fpls.2023.1186960)
Supplement: Supplementary file 1 [file DataSheet_1.docx]

Supplementary Material

Regulation of reactive oxygen species and phytohormones in osmotic stress tolerance during seed germination in *indica* rice

Ryusuke Kawaguchi, Chetphilin Suriyasak, Ryo Matsumoto, Yuta Sawada, Yuki Sakai, Norimitsu Hamaoka, Sasaki Kazuhiro, Koji Yamane, Yoichiro Kato, Christophe Bailly, Yushi Ishibashi

*** Correspondence:** Yushi Ishibashi, [yushi@agr.kyushu-ac.jp](mailto:yushi@agr.kyushu-ac.jp)

**Supplemental Figure 1.** Morphology of seeds of all cultivars and germinating phenotype of Rc348 and Rc10 under control and -1.5 MPa PEG conditions.

**Supplemental Figure 2.** Effects of DPI on Rc348 seed germination and exogenous H_2_O_2_ on Rc10 seed germination under -1.5 MPa PEG condition.

**Supplemental Figure 3.** Effects of exogenous sodium ascorbate (AsA) on seed germination and endogenous hormonal levels in Rc348 under -1.5 MPa PEG.

**Supplemental Table 1.** qRT-PCR primer sequences used in this study.


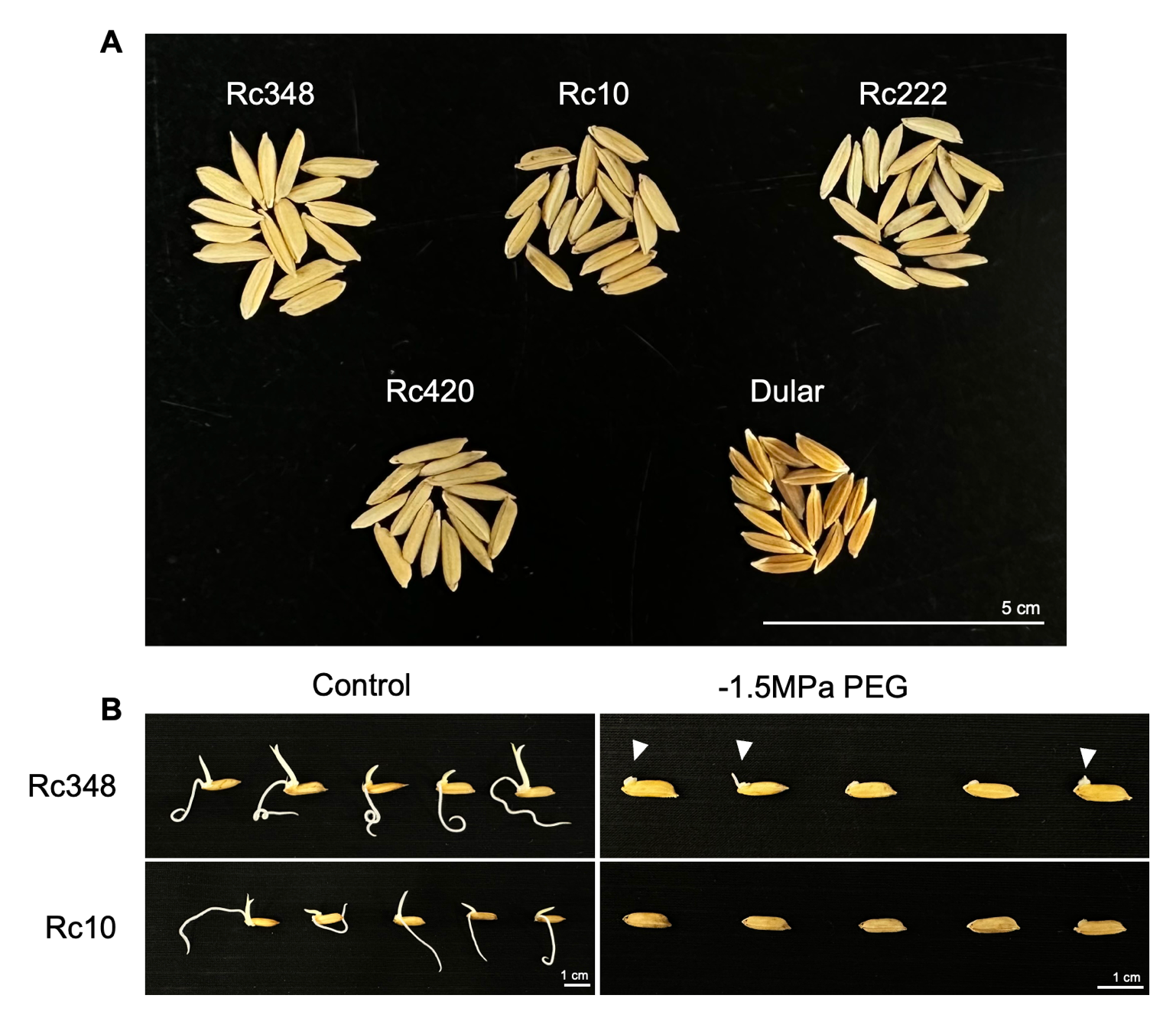


**Supplemental Figure 1. Morphology of seeds of all cultivars and germinating phenotype of Rc348 and Rc10 under control and -1.5 MPa PEG conditions.** (A) Seed morphology of all cultivars used in this study. (B) Germinating phenotype of Rc348 and Rc10 under control (non-stressed) and -1.5 MPa PEG condition at 96 HAI. White triangles show seed coat rupture and shoot emergence.

**
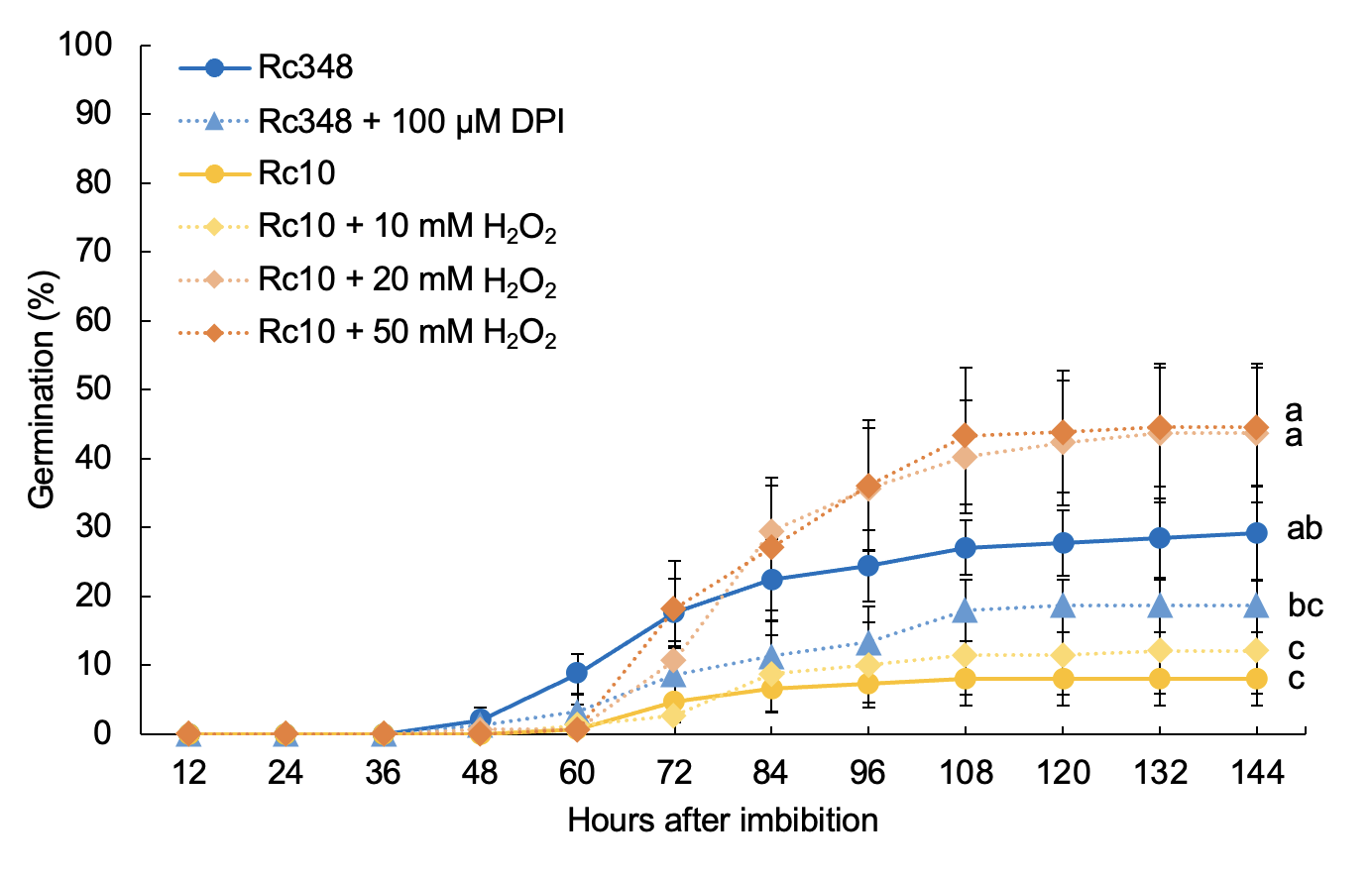
**

**Supplemental Figure 2. Effects of DPI on Rc348 seed germination and exogenous H_2_O_2_ on Rc10 seed germination under -1.5 MPa PEG condition.** Different letters show significant difference at *P* < 0.05 according to Tukey’s test (n=5) at 144 HAI.


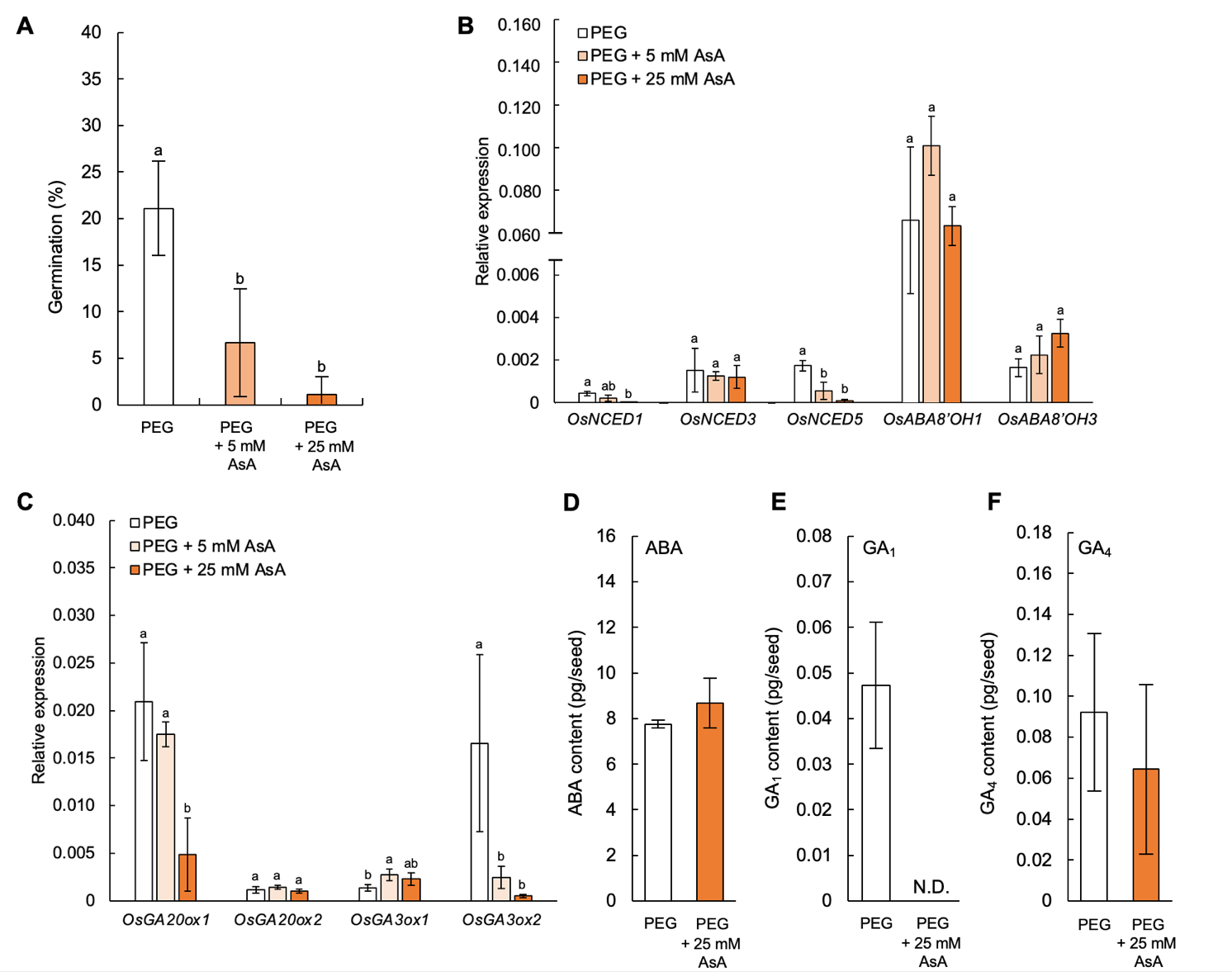


**Supplemental Figure 3. Effects of exogenous sodium ascorbate (AsA) on seed germination and endogenous hormonal levels in Rc348 under -1.5 MPa PEG.** (A) Germination percentage at 84 HAI of Rc348 under -1.5 MPa PEG only (PEG) or -1.5 MPa PEG with 5 mM and 25 mM of exogenous AsA. Relative expression of (A) ABA metabolism related-genes and (B) GA biosynthesis-related genes at 84 HAI. Endogenous (D) ABA, (E) GA_1_ and (F) GA_4_, in imbibed embryos at 84 HAI. Different alphabets show significant difference at *P* < 0.05 according to Tukey’s test. N.D., no detection.

# Supplementary Table 1. qRT-PCR primer sequences used in this study

| **qRT-PCR primers** | **Forward** | **Reverse** |
| --- | --- | --- |
| *OsActin*  (Os11g0163100) | GACTCTGGTGATGGTGTCAGC | GGCTGGAAGAGGACCTCAGG |
| *OsKAO*  (Os06g0110000) | GCTGAAGAGGGCAAATCCAAAGTGCAG | GAGGAACAAGTAGCCTAGCCTGGCTG |
| *OsGA20ox1*  (Os03g0856700) | CCACTACTTCCGGCGATTCTTCCAGCG | GACGTGGTCCTGGTGGAGGATGGTG |
| *OsGA20ox2*  (Os01g0883800) | CGCCGACTACTTCTCCAGCACC | GCTGTCCGCGAAGAACTCCCT |
| *OsGA3ox1*  (Os05g0178100) | GGAGAGCAAGGCCGTGTATCAGG | CTCTCCTTGTCCTCTTCCTTCGCTAC |
| *OsGA3ox2*  (Os01g0177400) | GAGTACATGGCCGTCCGCAAGAAG | GCGTGAGATCGAGGTAGCTAGTAGC |
| *OsNCED1*  (Os02g0704000) | CTGGAGCACATGGAGCTAGTGCACTCC | CCGACGCCGAAGTAGCCGTACCTG |
| *OsNCED3*  (Os03g0645900) | CCCCTCCCAAACCATCCAAACCGA | TGTGAGCATATCCTGGCGTCGTGA |
| *OsNCED5*  (Os12g0617400) | CACATCCGAGCTCCTCGTCGTGAAC | GAATCAAGAATCCATGGATGGCCGGTG |
| *OsABA8’OH1*  (Os02g0703600) | AAGTACAGGTGGTCCACGTCCAA | CCAGCTTAGCTGATGCTAGTATTC |
| *OsABA8’OH3*  (Os09g0457100) | AGTACAGCCCATTCCCTGTG | ACGCCTAATCAAACCATTGC |
| *OsGAMYB*  (Os01g0812000) | CAGTGGCAATTCATTCACTGAATC | TCCAGATCCCATTGAAGTGCTTTG |
| *OsSAPK8*  (Os03g0764800) | TAGTATGAGCAGCCAGTATGAGG | TCTTGTTGGTCGATGACTTACAT |
| *OsSAPK10*  (Os03g0610900) | CTGTTCTTCATTCGCAACCAAAA | ATCCTCAAAAGGATATGCACCAA |
| *OsAmy1A*  (Os02g0765600) | GATACGACGTCGAACACCTC | CGGATCGGATACAGCTCGTTG |
| *OsAmy1C*  (Os02g0765400) | TATCATGGAGGCTGACAGCG | GCTAATTGTGCCTCTCCACC |
| *OsAmy3B*  (Os09g0457400) | GATTGGGACACGGTATGACG | CTGCAGGAACTCTGAGACCG |
| *OsAmy3E*  (Os08g0473600) | GAGAAGGAAGGCCTCAGGGTTCCTG | GAAGCTCGCTCGTACACATCTCGCAG |
| *OsRbohA*  (Os01g0734200) | ATCCGCAAAATAAGCACCTCT | CAGTAGCCCATCACATCAAAGAC |
| *OsRbohB*  (Os01g0360200) | GGCTTCAATGCCTTCTGGT | ATGGCTCCTAAACAACCGA |
| *OsRbohC*  (Os05g0528000) | CCAGTGGGTGGGAAAAGTG | GTCCGATTGGCGGGTAAA |
| *OsRbohD*  (Os05g0465800) | CACAAGGTTATCGCACTGACG | AGCGATGAGTATGTTGGTTGA |
| *OsRbohE*  (Os01g0835500) | TGGTCTTGGAATTGGTGCTACTC | ACCATGTATGCTTTCCACCTCTTC |
| *OsRbohF*  (Os08g0453700) | CCTTTCTCCATCACTTCAGCA | GGGCCATCTACAAGCAACC |
| *OsRbohG*  (Os09g0438000) | GTCAAATGCTTATGCTGTCA | TGTCCAGTCTCCGTTTGTT |
| *OsRbohH*  (Os12g0541300) | TACTTCGGGCAGACACGGAT | GCGGGTTGCTGTCACTAAG |
| *OsRbohI*  (Os11g0537400) | ACCTTACCTGCGATTTTCCA | ACGAAGCAGTGGTGGGAGT |
|  |  |  |
